# HIV-1 Tat phosphorylation on Ser-16 residue modulates HIV-1 transcription

**DOI:** 10.1186/s12977-018-0422-5

**Published:** 2018-05-23

**Authors:** Andrey Ivanov, Xionghao Lin, Tatiana Ammosova, Andrey V. Ilatovskiy, Namita Kumari, Hatajai Lassiter, Nowah Afangbedji, Xiaomei Niu, Michael G. Petukhov, Sergei Nekhai

**Affiliations:** 1 Center for Sickle Cell Disease, Howard University, 1840 7th Street, N.W. HURB1, Suite 202, Washington, DC 20001 USA; 2 Department of Medicine, Howard University, Washington, DC USA; 3 Yakut Science Center for Complex Medical Problems, Yakutsk, Russia; 4 Division of Molecular and Radiation Biophysics, Petersburg Nuclear Physics Institute, Gatchina, Russia; 5 Research Center for Nanobiotechnologies, Peter the Great St. Petersburg Polytechnic University, St. Petersburg, Russia

## Abstract

**Background:**

HIV-1 transcription activator protein Tat is phosphorylated in vitro by CDK2 and DNA-PK on Ser-16 residue and by PKR on Tat Ser-46 residue. Here we analyzed Tat phosphorylation in cultured cells and its functionality.

**Results:**

Mass spectrometry analysis showed primarily Tat Ser-16 phosphorylation in cultured cells. In vitro, CDK2/cyclin E predominantly phosphorylated Tat Ser-16 and PKR—Tat Ser-46. Alanine mutations of either Ser-16 or Ser-46 decreased overall Tat phosphorylation. Phosphorylation of Tat Ser-16 was reduced in cultured cells treated by a small molecule inhibitor of CDK2 and, to a lesser extent, an inhibitor of DNA-PK. Conditional knock-downs of CDK2 and PKR inhibited and induced one round HIV-1 replication respectively. HIV-1 proviral transcription was inhibited by Tat alanine mutants and partially restored by S16E mutation. Pseudotyped HIV-1 with Tat S16E mutation replicated well, and HIV-1 Tat S46E—poorly, but no live viruses were obtained with Tat S16A or Tat S46A mutations. TAR RNA binding was affected by Tat Ser-16 alanine mutation. Binding to cyclin T1 showed decreased binding of all Ser-16 and Ser-46 Tat mutants with S16D and Tat S46D mutationts showing the strongest effect. Molecular modelling and molecular dynamic analysis revealed significant structural changes in Tat/CDK9/cyclin T1 complex with phosphorylated Ser-16 residue, but not with phosphorylated Ser-46 residue.

**Conclusion:**

Phosphorylation of Tat Ser-16 induces HIV-1 transcription, facilitates binding to TAR RNA and rearranges CDK9/cyclin T1/Tat complex. Thus, phosphorylation of Tat Ser-16 regulates HIV-1 transcription and may serve as target for HIV-1 therapeutics.

## Background

Complete eradication of HIV-1 virus in infected individuals is hindered by the presence of latent HIV-1 provirus, which is not affected by the existing anti-HIV-1 drugs [1]. Thus, novel approaches are needed to better understand and successfully target latent HIV-1 infection. HIV-1 transcription from HIV-1 LTR depends on both host cell factors and HIV-1 transactivation Tat protein [2]. HIV-1 Tat activates viral transcription by recruiting Positive Transcription Elongation Factor b (P-TEFb) that contains CDK9/cyclin T1 to TAR RNA [2]. Inability of Tat to recruit CDK9/cyclin T1 to TAR RNA may contribute to the establishment of latency [1]. Our earlier study showed that CDK2 phosphorylated HIV-1 Tat in vitro, although the phosphorylated residues were not clearly identified [3]. Subsequently, we found that Tat was phosphorylated in cultured cells and that the phosphorylation was significantly reduced when Ser-16 or Ser-46 residues were mutated [4]. Co-expression of Flag-tagged Tat S16A or Tat S46A mutants failed to activate integrated HIV-1 provirus with defective Tat [4]. We also showed that inhibition of CDK2 by iron chelators, 311 and ICL670, reduced Tat phosphorylation in cultured cells [5]. A recent study from Tyagi’s lab showed that Tat was phosphorylated in vitro by DNA-dependent protein kinase (DNA-PK) on Ser-16 and Ser-62 residues and that alanine mutations in these sites, separately or in combination, reduced HIV-1 replication [6]. HIV-1 Tat was also shown to be phosphorylated in vitro by a double-stranded RNA activated protein kinase R (PKR) on C-terminal residues [7, 8] and by protein kinase C (PKC) on Ser-46 [9]. PKR interacted with Tat in cultured cells [7] and phosphorylated Tat [8] or Tat-derived peptides [10] on C-terminal Ser-62, Thr-64 and Ser-68 residues. Phosphorylation of Tat by PKR enhanced Tat binding to TAR RNA and alanine mutations in Ser-62, Thr-64 and Ser-68 reduced Tat-mediated HIV-1 transcription activation [10]. In a recent study, PKR was shown to phosphorylate additional Tat residues including Thr-23, Thr-40, Ser-46, Ser-62 and Ser-68 in vitro [11]. In cultured cells, phosphorylation of Tat by PKR inhibited HIV-1 transcription by preventing the interaction of Tat with TAR RNA and reducing Tat translocation to the nucleus [11]. In addition to being phosphorylated, Tat was also shown to be methylated, acetylated and ubiquitinated (reviewed in [12]). Monoubiquitination of Tat on Lys-71 residue by Hdm2 increased Tat’s ability to activate HIV-1 transcription and did not lead to its degradation [13].

Here, we analyzed Tat phosphorylation in cultured cells using high resolution mass spectrometry. We detected with high confidence phosphorylation of Ser-16 residue, and with lower confidence phosphorylation of Ser-46, Thr-77, Ser-81, Thr-82 and Ser-87 residues. Using synthetic peptides that span several potential phosphorylation sites of Tat, we showed that CDK2/cyclin E predominantly phosphorylated Tat Ser-16 and that PKR predominantly phosphorylated Tat peptide containing Ser-46. Alanine mutations of either Ser-16 or Ser-46 decreased overall Tat phosphorylation. We used small molecule inhibitors of CDK2 and DNA-PK and high resolution mass spectrometry to explore the effect of CDK2 and DNA-PK inhibition on Tat Ser-16 phosphorylation in cultured cells. We developed conditional knock-downs of CDK2 and PKR in CEM T cells and tested them for HIV-1 replication which showed induction and inhibition of one round HIV-1 replication by PKR KD and CDK2 KD, respectively. To analyze functional consequences of Ser-16 and Ser-46 phosphorylation, we analyzed transcriptional activity of HIV-1 proviral DNA containing Ser-16 and Ser-46 alanine and phosphorylation-mimicking glutamic acid mutations which showed complete inhibition of transcription by alanine mutations and partial restoration of transcription by S16E mutation and poor restoration by S46E mutation. We also assembled pseudotyped viruses from mutant pNL4-3 Luc vectors that showed partial and weak compensation by Tat S16E and Tat S46E mutations, respectively. We were not able to assemble proviruses with Tat S16A or Tat S46A mutations. We also analyzed nuclear localization of Tat using EGFP-fused alanine and glutamic acid mutants of Ser-16 and Ser-46, which showed deficiency in nuclear localization for Tat S46E. Analysis of Tat ubiquitination showed no strong effects of Tat mutants on ubiquitination. TAR RNA binding was analyzed and found to be only affected by Tat S16A mutation. Analysis of Tat binding to cyclin T1 showed decreased binding of all mutants and Tat S16D and Tat S46D were having the strongest effect. Molecular modelling and molecular dynamic analysis revealed significant structural changes in Tat/CDK9/cyclin T1 complex with phosphorylated Ser-16, but not with phosphorylated Ser-46. Together, our results indicate Tat Ser-16 phosphorylation is an important event in HIV-1 transcription regulation and that it may facilitate binding to TAR RNA and the rearrangement of CDK9/cyclin T1/Tat complex.

## Results

### Analysis of HIV-1 Tat phosphorylation in cultured cells

**Fig. 1:**
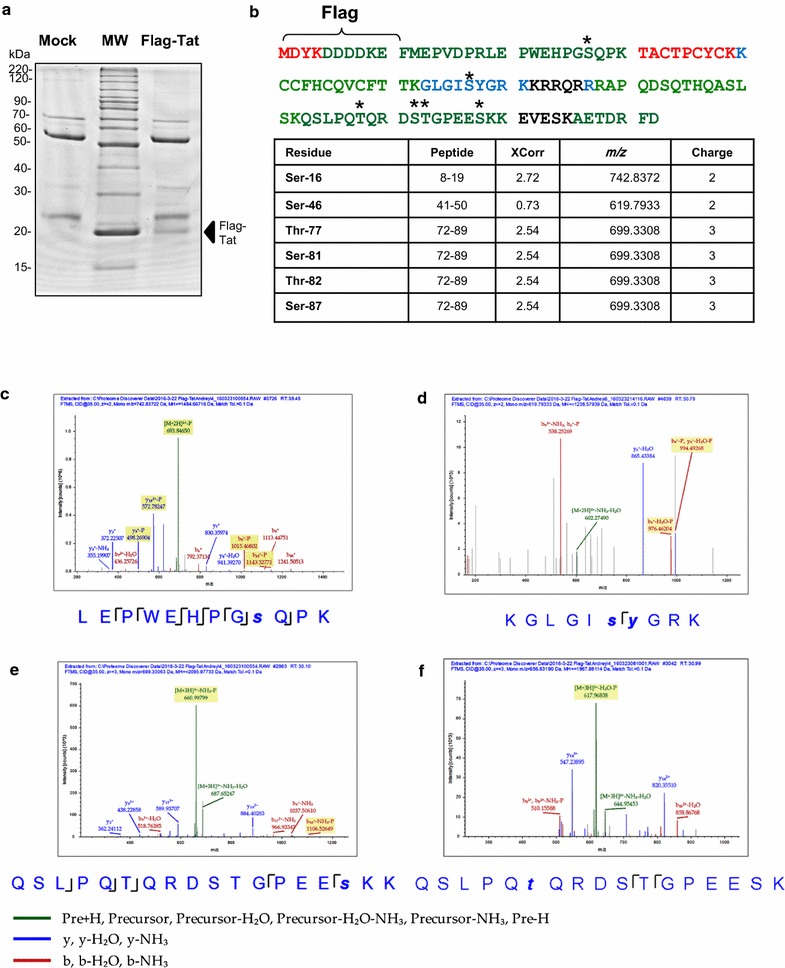
Tat phosphorylation in cultured cells. **a** Purification of Flag-tagged HIV-1 Tat for mass spectrometry analysis. Flag-tagged Tat was expressed in 293T cells, immunoprecipitated from cellular lysate with anti-Flag antibodies and resolved on 10% SDS-PAGE. Peptides were in-gel digested with trypsin, eluted and subjected to MS analysis on Thermo LTQ Orbitrap XL mass spectrometer. Position of Flag-Tat is shown. **b** MS/MS analysis of Tat phosphorylation. SEQUEST search results are shown for Tat peptides identified with high, median and low confidence indicated in green, blue and red, respectively. Peptides that were not detected are shown in black. Phosphorylated serine and threonine residues are marked by asterisks. Identified phosphopeptides are shown in the table. **c**–**f** Phosphopeptides spectra. **c** MS/MS spectra of the Ser-16 phosphorylated Tat peptide 8–19. **d** Ser-46/Tyr-47 phosphorylated Tat peptide 41–50. **e** Ser-81 phosphorylated Tat peptide 72–89. **f** Ser-87 phosphorylated Tat peptide 72–89. The colored peaks indicate matched MS/MS fragments. Green color indicates precursor ions. Blue and red colors indicate y and b ions, respectively

Our previous studies showed that Tat is phosphorylated in vitro and in cultured cells [3, 4]. Alanine mutations of serine residues located within S^16^QPR^19^ and S^46^YGR^49^ sequences of HIV-1 significantly reduced Tat phosphorylation suggesting that these residues might be phosphorylated in vivo [4]. Here, we analyzed phosphorylation of HIV-1 Tat expressed in cultured cells using high resolution mass spectrometry. Flag-Tat was expressed in 293T cells which were briefly treated with 0.1 M okadaic acid prior to Tat isolation by immunoprecipitation with anti-Flag antibodies and purification on SDS PAGE (Fig. 1a). Tat was in gel digested with trypsin (see Methods) and subjected to LC-MS/MS analysis (Fig. 1b). Recovered Tat-derived peptides depicted 90% coverage of the Flag-Tat sequence and included potential phosphorylation sites (Fig. 1b). Analysis of posttranslational modifications in Tat showed multiple phosphorylation sites on serine and threonine residues including Ser-16, Ser-46, Thr-77, Ser-81, Thr-82 and Ser-87 (Fig. 1b–f). While signal for phospho Ser-16 containing peptide was relatively high (~ 10^6^), signals for all other phosphorylated peptides including GLGIsyGR peptide that contains Ser-46 were significantly lower (~ 10^3^) (Fig. 1c–f) suggesting that Ser-16 was the major phosphorylation site and that all other sites had much lower levels of phosphorylation. Thus, mass spectrometry analysis identified the N-terminal Ser-16, Ser-46 and several C-terminal residues of Tat being phosphorylated in vivo.

### CDK2 phosphorylates Tat Ser-16 in vitro

**Fig. 2:**
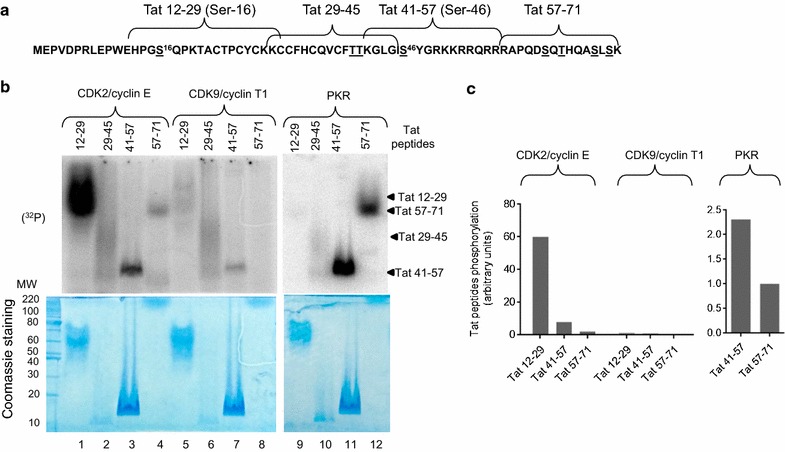
CDK2 phosphorylates Tat Ser-16 and PKR phosphorylates Tat Ser-46 in vitro. **a** Sequence of Flag-tagged HIV-1 Tat indicating peptides that were used for the analysis of Tat phosphorylation in vitro. Potential phosphorylation sites are underscored. Tat Ser-16 and Ser-46 residues are further indicated with superscript numbering. Ser-16 (peptide 12–29); Thr-39 and Thr-40 (peptide 29–45); Ser-46 (peptide 41–57); and Ser-62, Thr-64, Ser-68 and Ser-70 (peptide 57–71). **b** Phosphorylation of Tat peptides in vitro. Upper panels, Tat peptides (4 µg) were phosphorylated in vitro with recombinant enzymes CDK2/cyclin E (lanes 1–4), CDK9/cyclin T1 (lanes 5–8), and PKR (lanes 9–12) with γ(^32^P)ATP as described in Methods. Phosphorylated peptides were resolved on 12% SDS Tris-Tricine gel containing 6 M urea, stained with SimpleBlue SafeStain (Coomassie), dried and analyzed by Phospho Imager. Phosphorylated Tat peptides position indicated with arrows on the right. Lower panel, Coomassie stained gel of Tat peptides showing 20 μg of Tat peptides 12–29, 29–45 and 57–71 and 2 μg of Tat peptide 41–57 resolved on 12% SDS Tris-Tricine gel with urea and stained with SimpleBlue SafeStain (Coomassie). **c** Relative intensities of the peptides phosphorylated by CDK2/cyclin E, CDK9/cyclin T1 or PKR were quantified with OptiQuant software (Packard)

We further analyzed HIV-1 Tat phosphorylation in vitro by CDK2 and PKR using Tat peptides that span potential phosphorylation sites including Ser-16 (residues 12–29); Thr-39 and Thr-40 (residues 29–45); Ser-46 (residues 41–57); and Ser-62, Thr-64, Ser-68 and Ser-70 (residues 57–71) (Fig. 2a). Tat-derived peptides were resolved on SDS PAGE containing 6 M urea and stained with Coomassie (Fig. 2b, lower panels). Tat peptide 12–29 contains Ser-16 and also four cysteine residues that resulted in its aggregation despite the addition of 1 mM DTT and sonication. This peptide migrated as a major band at 70 kDa (Fig. 2b, lower panel, lane 1). Peptide 29–45 also aggregated and formed a smear on a Coomassie stained gel (Fig. 2b, lower panel, lane 2). Peptide 41–57 containing Ser-46 was fully soluble and migrated with the front (Fig. 2b, lower panel, lane 3). Peptide 57–71 could not be seen on a Coomassie stained gel even when loaded at high amount (20 μg) on the gel (Fig. 2b, lower panel, lane 4). Recombinant enzymes CDK2/cyclin E, CDK9/cyclin T1 and PKR were used for in vitro phosphorylation of Tat peptides. Recombinant CDK2/cyclin E primarily phosphorylated a peptide containing Ser-16 (Fig. 2b, lane 1) and to a lesser extent peptides containing Thr-39/Thr-40 and Ser-46 (Fig. 2b, lanes 3–4; see quantifications in Fig. 2c). Recombinant CDK9/cyclin T1 showed low levels of phosphorylation of peptides containing Ser-16, Thr-39/Thr-40 and Ser-46 (Fig. 2b, lanes 5 and 7; and Fig. 2c). PKR phosphorylated Tat 41–57 peptide containing Ser-46 (Fig. 2b, lane 11), and also strongly phosphorylated Tat 57–71 peptide containing C-terminal serine and threonine residues (Fig. 2b, lane 12). Quantification analysis showed that PKR primarily phosphorylated Ser-46 containing peptide (Fig. 2c).

**Fig. 3:**
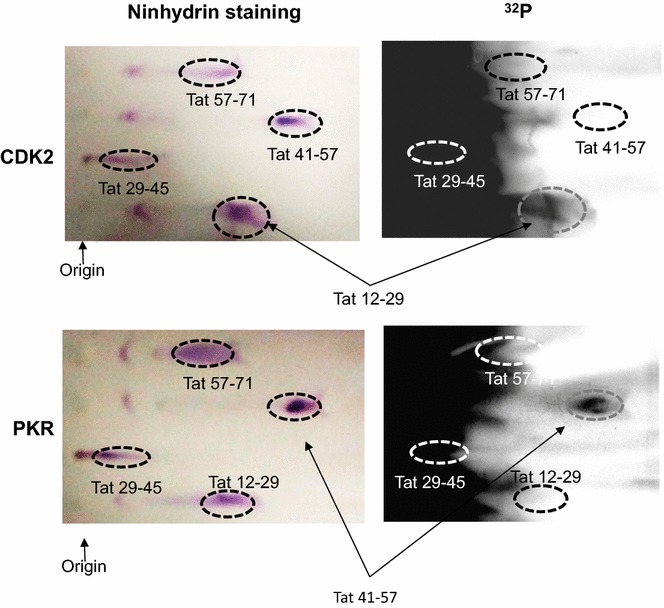
Hunter peptide mapping analysis of Tat phosphorylation by CDK2 and PKR. Tat-derived peptides were phosphorylated in vitro by CDK2/cyclin E or PKR, as indicated. The reactions were loaded on nitrocellulose plates and peptides were resolved by thin layer electrophoresis as described in Methods. Plates were dried and stained with ninhydrin (left panels) or exposed to Phospho Imager screen (right panels). Origin and peptide positions are indicated on figure. The results are representative from 2 experiments

We further verified that Tat Ser-16 containing peptide is phosphorylated by CDK2/cyclin E using Hunter peptide thin layer electrophoresis system that resolves (^32^P) peptides. We previously utilized this technique for the analysis of CDK9 phosphorylation [14]. Tat peptides were phosphorylated by CDK2/cyclin E and PKR in vitro and resolved on Hunter peptide mapping system. Then their positions were determined by ninhydrin staining (Fig. 3, left panels). Peptide phosphorylation detected by PhosphoImager showed that CDK2/cyclin E phosphorylated Tat 12–29 peptide containing Ser-16 (Fig. 3) and PKR phosphorylated peptide Tat 41–57 containing Ser-46 (Fig. 3). We could not detect phosphorylation of Tat 29–45 as this peptide migrates with the (^32^P) ATP containing front. Taken together, the Tat peptides phosphorylation analysis indicated that Tat Ser-16 was the major phosphorylation site for CDK2/cyclin E and Tat Ser-46—for PKR.

### Tat phosphorylation in vivo

**Fig. 4:**
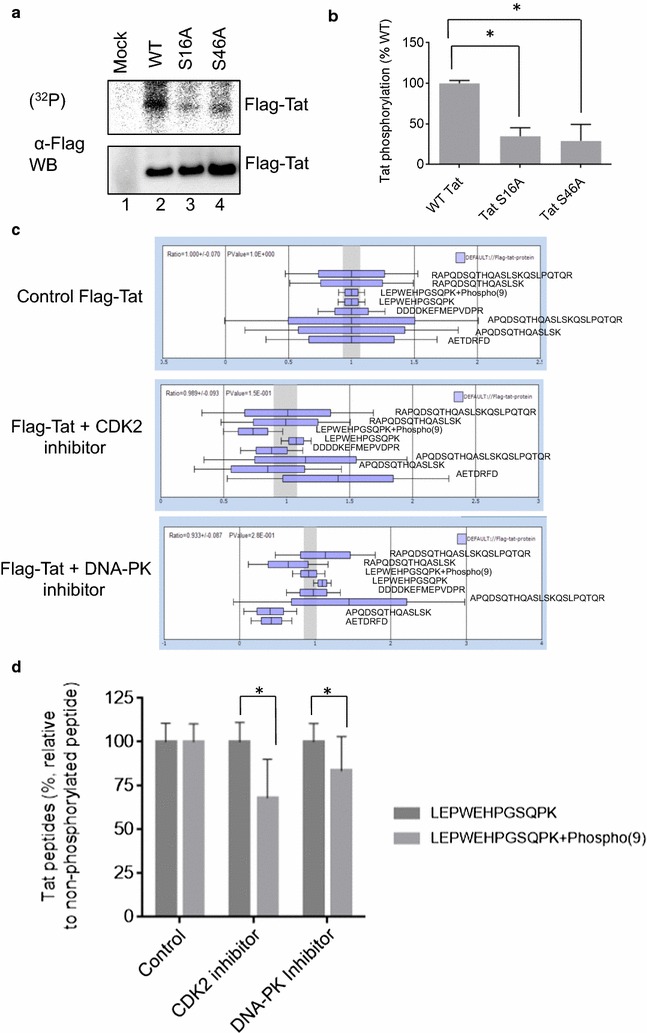
Tat phosphorylation and the effect of CDK2 and PKR in cultured cells. **a** Mutation of Ser-16 or Ser-46 residue reduced HIV-1 Tat phosphorylation in cultured cells. Flag-tagged Tat, WT and S16A and S46A mutants were expressed in 293T cells and metabolically labeled with (^32^P) orthophosphate. Tat protein was immunoprecipitated from cell lysates, resolved on 10% SDS-PAGE and exposed to Phosphor Imager screen. Tat expression was verified by Western blotting with anti-Flag antibodies. Lane 1, mock-transfected cells. Lane 2, WT Tat. Lane 3, Tat S16A mutant. Lane 4, Tat S46A mutant. The figure represents one of the three independent experiments. **b** Relative intensities of Tat and the mutants phosphorylation from three independent experiments. The mean ± SD are shown. *p < 0.01. **c** Label-free quantitative analysis of the high resolution MS spectra produced by Orbitrap MS scans for Tat by SIEVE 2.1 software. Average intensities of the indicated Tat peptide are shown with mean and standard deviations. **d** Quantification of non-phosphorylated and Ser-16 phosphorylated LEPWEHPGSQPK + Phospho(9) peptides derived from the data on **c**. Data are further adjusted to indicate the ratio of non-phosphorylated versus phosphorylated peptides. *p < 0.05

To confirm phosphorylation of Tat in vivo, Flag-tagged WT Tat, Tat S16A and Tat S46A mutants were expressed in 293T cells. The cells were metabolically labeled with (^32^P)-orthophosphate for 3 h and Tat was immunoprecipitated with anti-Flag antibodies (Fig. 4a). Mutation of either Ser-16 or Ser-46 reduced the overall Tat phosphorylation levels (Fig. 4a, compare lane 2 to lanes 3 and 4; see quantification in Fig. 4b), in accordance with our previous findings that alanine mutations of Tat Ser-16 and Tat Ser-46 prevented Tat phosphorylation [4].

As Tat Ser-16 can be phosphorylated by CDK2 as shown here and also by DNA-PK as determined by Tyagi’s group [6], we tested the effect of CDK2 and DNA-PK small molecule inhibitors on Tat phosphorylation in cultured cells. Flag-Tat was expressed in 293T cells and the cells were treated overnight with SU 9516, an inhibitor of CDK2 (Tocris) or NU 7441, an inhibitor for PK DNA (Tocris). After the overnight treatment, all cells were also briefly treated with 0.1 M okadaic acid to induce Tat phosphorylation. Tat was immunoprecipitated from cellular lysates with anti-Flag antibodies and separated on SDS PAGE as described above. Tat containing gel pieces were in gel digested with trypsin and subjected to LC-MS/MS analysis. Mass spectra were analyzed with Proteome Discoverer 1.4 and quantified using a label-free approach. We used SIEVE 2.1 software which reanalyzes the original MS spectra and extracts selected ions (frames) with the highest ion current values and integrates their ion elution profiles. We focused on Tat Ser-16 peptide that was previously identified with high confidence. Using trend analysis, we detected over 700 frames which were matched by importing Proteome Discoverer 1.4 analysis results back into SIEVE 2.1. We detected eight Flag-Tat peptides (Fig. 4b) which were normalized using global normalization to equal ratios for each experimental group. Relative ratios of the Tat peptides (Fig. 4c) indicate that non-phosphorylated LEPWEHPGSQPK peptide containing Ser-16 was present at similar ratios in the control and the treatment samples. In contrast, ratios of Ser-16 phosphopeptide (LEPWEHPGSQPK + Phospho (9)) were decreased in the samples treated with CDK2 and DNA-PK inhibitors suggesting decreased Ser-16 phosphorylation. Quantification of the non-phosphorylated peptide (LEPWEHPGSQPK) and phospho Ser-16 peptide (LEPWEHPGSQPK + Phospho (9)) showed about 30% decrease in CDK2-inhibitor treated cells and about 15% decrease in DNA-PK inhibitor treated cells (Fig. 4d). Thus both CDK2 and DNA-PK can phosphorylated Tat Ser-16 in vivo and CDK2 is likely to be the main contributor.

### Effect of CDK2 and PKR knock downs on HIV-1 replication

**Fig. 5:**
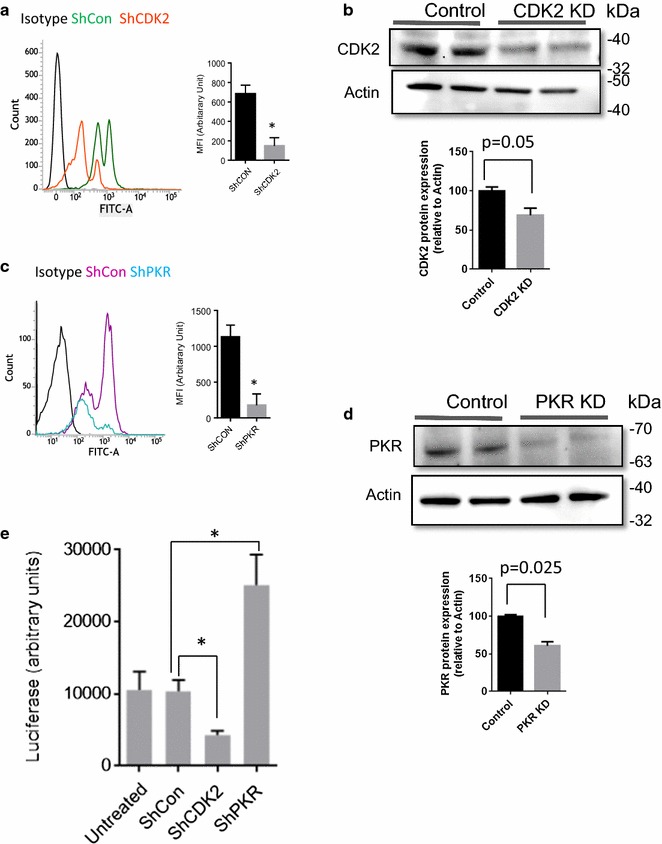
Effect of CDK2 and PKR Knock downs on HIV-1 replication. **a**–**d** CDK2 and PKR knockdown were generated in CEM T cells stably transduced with lentiviruses expressing CDK2 or PKR-targeting shRNA, or control non-targeting shRNA. **a**, **c** Expression of PKR and CDK2 in CEM T cells respectively determined by FACS analysis. Representative histogram shows isotype antibody staining (black), shRNA control (green or purple respectively) and CDK2 or PKR-targeting shRNA (orange or blue respectively). Bar graph of mean fluorescent intensity (MFI, y axis starts at mean fluorescence intensity of the isotype control; n = 2 per group).Mean ± SD. **b**, **d** Protein expression levels of CDK2 and PKR in stable knock down cell lines. Actin was used as normalization control. Bar graphs show the extent of the corresponding protein knock outs normalized to actin. **e** CEM T cells were infected with HIV-1-LUC-G virus and luciferase activity was measured at 48 h post infection. The mean ± SD are shown. *p ≤ 0.01

To confirm that CDK2 and PKR were critical for HIV-1 replication, we generated stable shRNA-mediated knock downs (KD) for CDK2 (Fig. 5a, b) and PKR (Fig. 5c, d) in CEM T cells. Protein levels of CDK2 and PKR were reduced when measured by flow cytometry (Fig. 5a, c) or by Western blot (Fig. 5b, d). To test the effect of knockdowns on HIV-1 replication, CEM T cells were infected with Vesicular stomatitis virus G protein (VSV-G)**-**pseudotyped pNL4-3.Luc.R-E-virus (HIV-1-LUC-G). The CDK2 KD inhibited HIV-1 replication (Fig. 5e) in agreement with our recent report showing reduction of one round HIV-1 infection in CDK2 KD cells [15]. In contrast, one round of HIV-1 infection was induced in the PKR KD cells (Fig. 5e) in agreement with a recent study that showed inhibition of HIV-1 replication by PKR [11].

### Tat Ser-16 alanine mutation inhibits HIV-1 transcription and Tat S16E mutation restores it

**Fig. 6:**
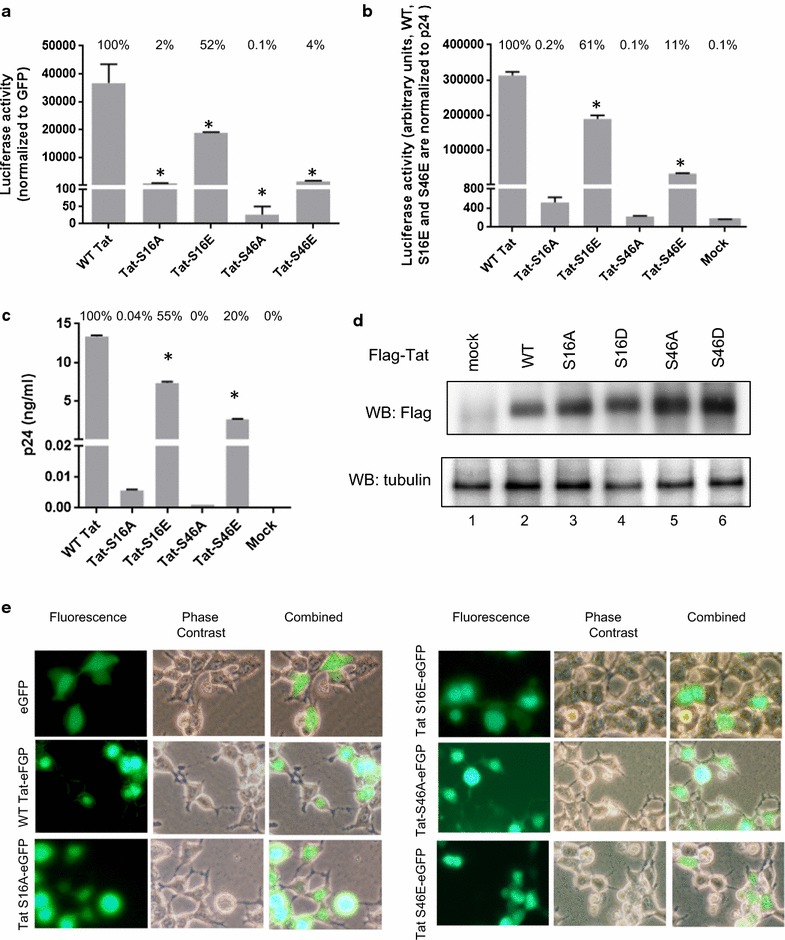
Alanine mutations in Tat Ser-16 and Ser-46 reduced HIV-1 proviral DNA transcription replication. **a** Transcription activity of pNL4-3.Luc.R-E-vectors with WT and mutant Tat. 293T cells were transfected with the indicated proviral vectors and also co-transfected with EGFP expressing vector. At 48 h posttransfection, the cells were lysed and luciferase activity was detected. EGFP fluorescence was measured and used for normalization. Results are averages of quadruplicates from a typical experiment of 3 performed. Percent of activity relative to the WT Tat are shown above the bars. *p ≤ 0.001. **b**, **c** Replication of VSVg pseudotyped pNL4-3.Luc.R-E-vectors with WT and mutant Tat S16A, S16E, S46A and S46E sequences. 293T cells were transfected with proviral vectors and VSVg-expressing plasmid. At 48 h posttransfection, media was collected and used to infect CEM T cells (**b**) and for p24 measurement (**c**). In **b**, luciferase activity for WT, S16E and S46E viruses was normalized to p24. Percent of activity relative to the WT Tat is shown above the bars. *p ≤ 0.001. **d** Expression of Flag-tagged WT Tat and mutants. 293T cells were transfected with Flag-Tat vectors expressing WT Tat, Tat S16A, Tat S16D, Tat S46A and Tat S46D mutants. At 48 h posttransfection, the cells were lysed. The lysates were resolved on the 12% SDS-PAGE and immunoblotted with anti-Flag (upper panel) or anti-tubulin (low panel) antibodies. **e** 293T cells were transfected with vectors expressing EGFP, WT Tat-EGFP, Tat S16A-GEFP, Tat S16E-EGFP, Tat S46A-EGFP and Tat-S46E-EGFP. At 24 h posttransfection the cells were photographed on Olympus IX51 using a blue filter for EGFP fluorescence with × 300 magnification

Next, we analyzed the effect of Tat Ser-16 and Ser-46 mutations on HIV-1 transcription from proviral DNA. We introduced Tat alanine and phosphorylation-mimicking glutamic acid mutations in pNL4-3 Luc proviral vector which was transfected in 293T cells along with EGFP expressing vector to monitor transfection efficiency. Both Tat S16A and Tat S46A mutations strongly reduced luciferase expression to 2 and 0.1% respectively, suggesting inhibition of HIV-1 transcription (Fig. 6a). Tat S16E mutation showed minimal effect (52%, Fig. 6a) suggesting that glutamic acid substitution supported HIV-1 transcription that was inhibited by the alanine mutation. In contrast, Tat S46E mutation only demonstrated minimal 4% transcription efficiency comparing to WT Tat (Fig. 6a) suggesting that any alteration of in Tat Ser-46 residue is inhibitory and that phosphorylation of Ser-46 is also likely to suppress HIV-1 transcription. To further analyze the effect of Tat Ser-16 and Ser-46 mutations, we generated pseudotyped HIV-1 viruses using pNL4-3 Luc proviral DNA and VSVG-expressing vector and then infected CEM T cells to analyze one round HIV-1 infection. We could only assemble viruses with Tat S16E or Tat S46E mutations and not with Tat S16A or Tat S46A mutations (Fig. 6b). Thus we normalized p24 levels for Tat S16E and Tat S46E viruses but not for Tat S16A and Tat S46S viruses that produced background levels of p24 (Fig. 6b, c). Thus, viruses assembled from proviral vectors with Tat S16E or Tat 46E mutations led to generation of replication capable viruses (Fig. 6b). The virus with Tat S16E mutation replicated well (61% comparing to WT Tat, Fig. 6b) and had a robust p24 production during the viral assembly (55%, Fig. 6c). In contrast, the virus with Tat S46E did not replicate well (11% comparing to WT, Fig. 6b) and also showed less robust p24 production during viral assembly (20%, Fig. 6c), suggesting that Tat Ser-46 phosphorylation can have a negative effect on viral replication. To analyze whether Tat mutants expressed well, WT and mutant Tat were expressed as Flag fusions in 293T cells. All tested mutants expressed well (Fig. 6d) suggesting that HIV-1 transcription and replication defects were not due to the deficiency in Tat production.

We next tested the effect of Tat mutations on Tat nuclear localization which was shown to be affected by Tat Ser-46 phosphorylation [11]. EGFP-fused WT Tat and mutants were expressed in 293T cells and examined under fluorescent microscope. WT Tat and all mutants except Tat S46E were localized in the nucleus (Fig. 6e). However, Tat S46E showed more diffused localization similar to EGFP suggesting that it may have a defect. Taken together, the phosphomimetic mutation of Tat Ser-16 restored HIV-1 transcription and viral replication, while alanine and phosphomimetic mutation in Tat Ser-46 led to a complete or partial inhibition of HIV-1 transcription and replication.

### Effect of Tat phosphorylation on its ubiquitination

**Fig. 7:**
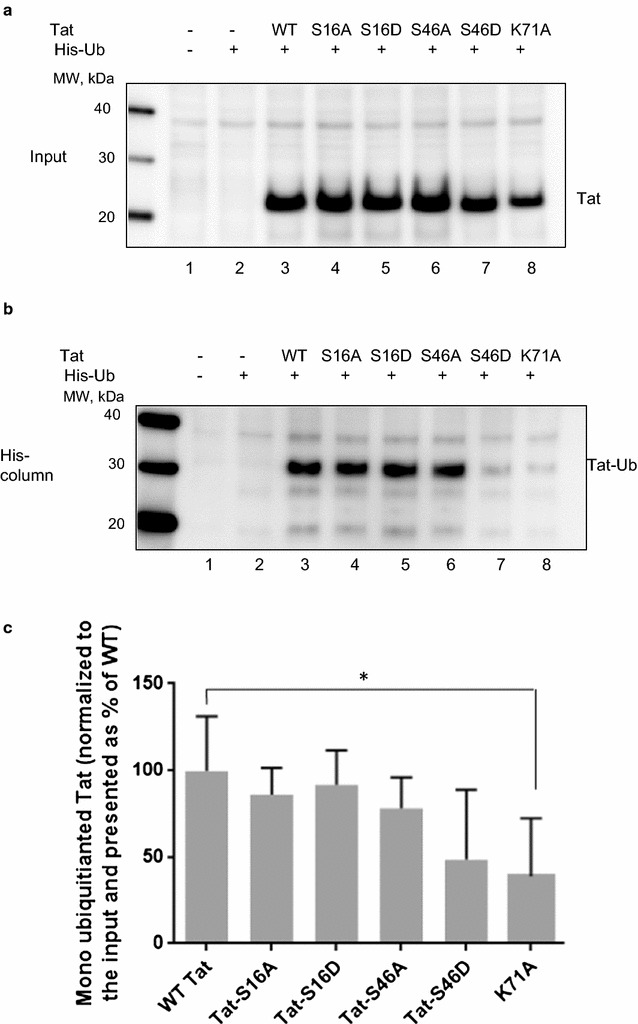
Tat Ser-46 mutations on its ubiquitination. **a** Tat S46A mutation decreased Tat ubiquitination. 293T cells were co-transfected with pCI-His-hUbi plasmid and WT Flag-Tat, Flag-Tat S16A, Flag-Tat S46A expressing plasmids. The cells were lysed at 48 h posttransfection. His–Ub conjugated proteins were extracted in guanidine denaturing buffer and purified on Ni–NTA agarose beads as described in Methods. Proteins were eluted from the beads and resolved on 12% Tris-Tricine SDS-PAGE, transferred to polyvinylidene fluoride (PVDF) membranes and immunoblotted with anti-Flag antibodies which detected monoubiquitinated Tat. Loading controls were obtained by resolving a portion of the total lysate on 12% SDS-PAGE. Lower panel show quantification as a mean of three independent measurements ± SD. Unpaired *t* test was used to test statistical significance. *p ≤ 0.01. **b** Tat S46D mutation decreased Tat ubiquitination. 293T cells were transfected and processed as in **a** except WT Flag-Tat, Flag-Tat S16D, Flag-Tat S46D and Flag-Tat K71A expressing plasmids were used. Lower panel show quantification

We next analyzed whether Tat phosphorylation had an effect on its ubiquitination, since Tat was previously shown to be monoubiquitinated [13] and because ubiquitination is generally mediated by protein phosphorylation [16]. To analyze ubiquitination of Tat, we expressed Flag-tagged Tat along with His-tagged Ubiquitin (His-Ub) in 293T cells (Fig. 7a). We separated His-Ub on a Ni column and quantified Tat ubiquitination using anti-Flag antibodies (Fig. 7b). None of the Tat mutations, except Tat S46D and previously reported Tat K71A mutant [13], showed reduced ubiquitination (Fig. 7b). However, we did not achieve statistical significance for Tat 46D in four separate experiments (Fig. 7c) suggesting that this reduction is moderate. Both Tat S16A and Tat S16D mutants remained monoubiquitinated (Fig. 7a, b) suggesting that Tat Ser-16 phosphorylation has no effect on ubiquitination. Collectively, Tat ubiquitination was mostly non-affected by Ser-16 or Ser-46 mutations, suggesting no effect of Tat phosphorylation on Ubiquitination.

### Tat S16A mutation reduced Tat binding to TAR RNA

**Fig. 8:**
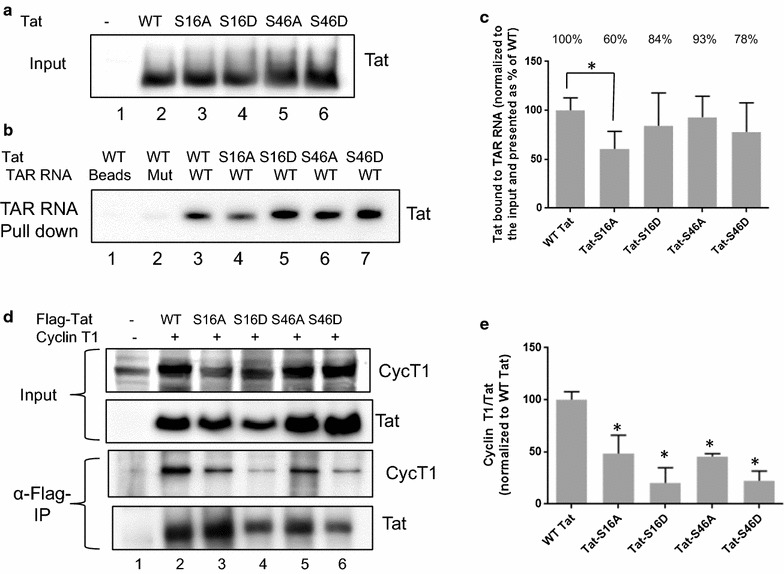
Effect of Tat S16A, S16D, S46A and S46D mutations on the interaction with TAR RNA and association with cyclin T1. **a**–**c** Tat Ser-16 and Ser-46 mutations decrease the interaction of Tat with TAR RNA. 293T cells were transfected with plasmids expressing WT Flag-Tat, Flag-Tat S16A or Flag-Tat S46A. The cells were lysed at 48 h posttransfection and the lysates were incubated with WT TAR RNA and mutant TAR RNA lacking bulge and immobilized on streptavidin beads. The beads were washed and proteins were eluted with SDS loading buffer and resolved on the 12% SDS-PAGE. Tat and TAR RNA were detected with anti-Flag and anti-biotin antibodies, respectively. **a** Tat loading control. Lane 1, control minus Tat. **b** Tat bound to the TAR RNA beads. Lane 1, control beads with no TAR RNA. Lane 2, control with mutant TAR RNA. **c** Quantification of Tat bound to TAR RNA beads relative to the loading control with asterisk indicating *p *≤ 0.01. **d**, **e** Tat Ser-16 and Ser-46 mutations decreased Tat association with cyclin T1. 293T cells were transfected with plasmids expressing WT Flag-Tat, Flag-Tat S16A, Flag-Tat S16D, Flag-Tat S46A and Flag-Tat S46D and with cyclin T1 expressing vector. The cells were lysed at 48 h posttransfection. Tat was immunoprecipitated with anti-Flag antibodies from the lysates and proteins were resolved on the 12% SDS-PAGE. Tat and cyclin T1 were detected with anti-Flag and anti-cyclin T1 antibodies. **e** Quantification cyclin T1/Tat ratio adjusted to the WT Tat control. Mean of three independent measurements ± SD are shown.*p ≤ 0.001

Previously, Tat phosphorylation in vitro by PKR was shown to enhance [10] as well as inhibit [11] its interaction with TAR RNA. Thus, we analyzed the effect of Tat Ser-16 and Ser-46 mutations on the binding of Tat to TAR RNA. Biotinylated 58 nucleotides long TAR RNA was coupled to the avidin-containing agarose beads and incubated with cell lysates prepared from cells transfected with Flag-Tat expressing vectors. All mutants as well as WT Tat were well expressed (Fig. 8a). Tat associated with TAR RNA bound to the beads was resolved on SDS-PAGE and detected with anti-Flag antibodies. Tat did not bind to the mutant TAR RNA that lacked the bulge structure (Fig. 8b, lane 2). Tat S16A mutant had a reduced binding to TAR RNA (40% reduction, Fig. 8c). Tat S46D mutant showed some reduction in TAR RNA binding but it was not statistically significant (Fig. 8c). Both Tat S16D and Tat S46A mutants bound to TAR RNA (Fig. 8c). Thus, Tat Ser-16 phosphorylation enhanced binding of Tat binding to TAR RNA.

### Tat S16D and Tat S46D mutations reduced Tat binding to CDK9/cyclin T1

To analyze the effect of Tat phosphorylation on the binding to CDK9/cyclin T1, we expressed Flag-tagged Tat and Tat S16A, S16D, S46A and S46D mutants along with cyclin T1 and then immunoprecipitated Tat with anti-Flag antibodies. All mutants bound less cyclin T1 (Fig. 8d, e), but Tat S16D and Tat S46D mutants showed the strongest reduction in cyclin T1 binding (Fig. 8d, lanes 4 and 6, and Fig. 8e). These results suggest that Ser-16 and Ser-46 phosphorylation might affect the interaction of Tat with CDK9/cyclin T1 complex.

### Molecular dynamics analysis of the effect of Tat Ser-16 and Ser-46 phosphorylation on Tat/CDK9/cyclin T1 complex

To further understand how the Tat Ser-16 and Ser-46 residues influence Tat binding to CDK9/cyclin T1, computational approach was used in which the residues were modeled in CDK9/cyclin T1/Tat complex with ICM-Pro software package using the coordinates of PDB entry 3MIA [17] as the template. To elucidate structural consequences of Tat phosphorylation at Ser-16 and Ser-46, we performed 20 ns Molecular Dynamics (MD) simulations in a periodical water box for the CDK9/Cyclin T1/Tat protein complexes in which Tat was non-phosphorylated on Ser-16 and Ser-46 (S16&S46) or double phosphorylated (S16P&S46P). Since CDK9 Thr-186 is phosphorylated in the kinase active P-TEFb complex [18], a phosphate group was introduced into this residue as well in all cases (Tpo186). Initial conformations of all these complexes were identical, except for the side chains of phosphorylated Ser-16 and Ser-46 residues obtained by global energy minimization using standard ICM-Pro protocol.

**Fig. 9:**
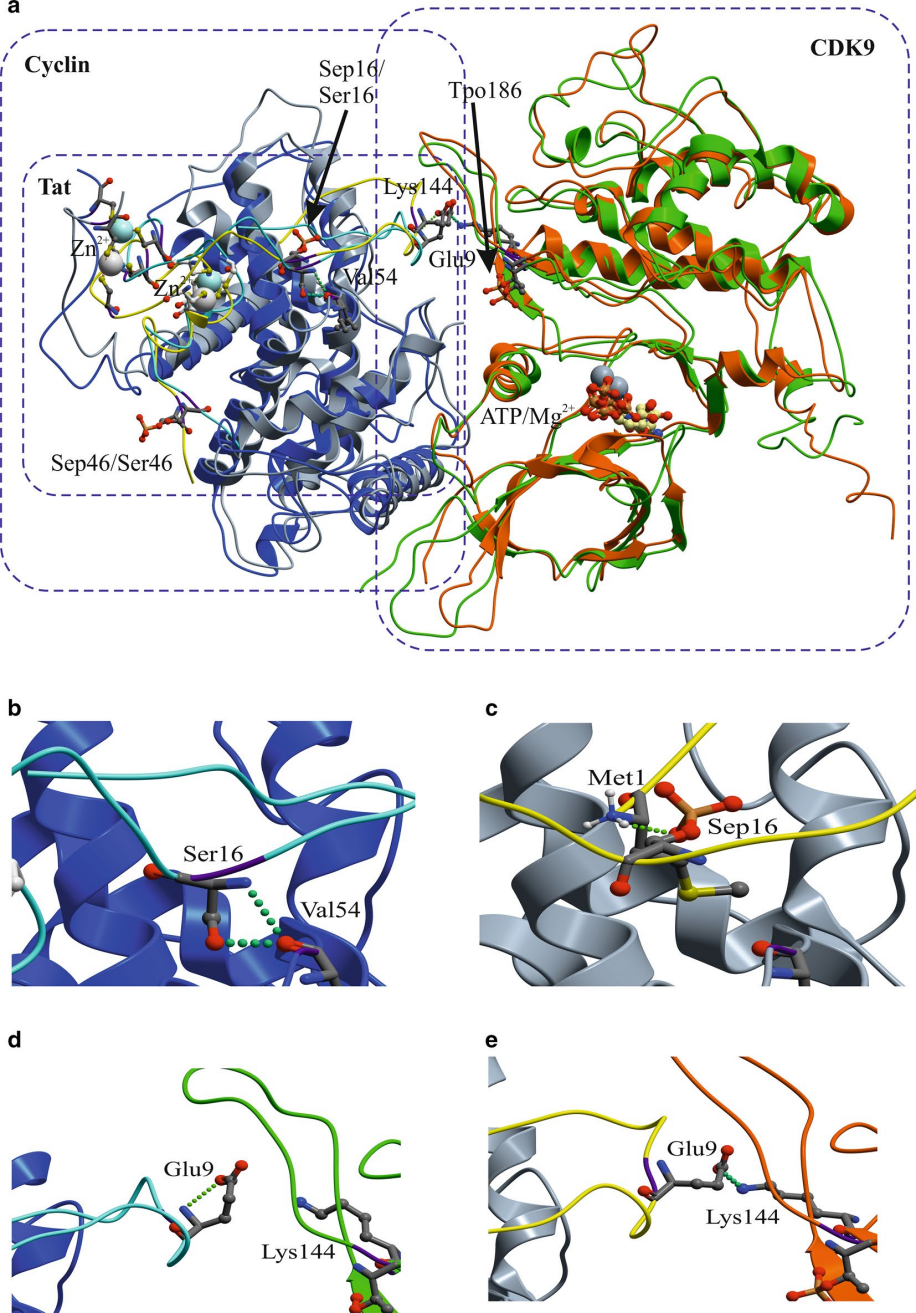
Molecular dynamics analysis (MD) of CDK9/Cyclin T1/Tat complex with phosphorylated Tat Ser-16 and Ser-46. **a** Spatial superposition of the CDK9/Cyclin T1/Tat complexes with non-phosphorylated Tat (S16&S46) and Tat phosphorylated on Ser-16 and Ser-46 (S16P&S46P) at 20 ns MD time, protein main chains are shown as follows: Cyclin T1—in blue and grey colors, CDK9—in green and carrot colors and Tat—in yellow and cyan colors, respectively. CDK9 Thr-186 was phosphorylated in all the MD simulations. ATP^+^Mg2^+^ is shown in ball-and-stick presentation. Initial conformation of the complexes were built by homology with crystal structure (PDB: 3MIA) as described in Methods. Arrows indicates the position of Ser-16 and phosphorylated Ser-16 (Sep16) and phosphorylated Thr 186 (Tpo186). Phosphorylated Ser-46 is abbreviated as Sep46. b Enlarged picture showing interaction of Tat Ser-16 with Cyclin T1 Val-54. **b**, **c** Enlarged pictures showing conformational change of Tat protein due to its phosphorylation. Initial interaction of Tat Ser-16 with Cyclin T1 Val-54 (**b**) is lost upon Ser-16 phosphorylation and the interaction with Tat Met-1 is formed (**c**). **d**, **e** Enlarged pictures showing interaction of Tat Glu-9 with CDK9 Lys-144 when Tat was phosphorylated (**e**) and no interaction when Tat was not phosphorylation (**d**)

Figure 9a shows spatial superposition of the S16&S46 and the S16P&S46P complexes at the final point of the 20 ns MD trajectory. Analysis of the CDK9/cyclin T1/Tat complex crystal structure (PDB: 3MIA) as well as the results of 20 ns MD showed that the side chain of Ser-16 is buried in the Tat-Cyclin T1 interface and its conformation is stabilized by the intermolecular hydrogen bond with the main chain oxygen of Cyclin T1’s Val-54. This hydrogen bond was found to be remarkably stable in the S16&S46 complex and preserved throughout the entire 20 ns of MD time.

Unlike Ser-16, Ser-46 is exposed to solvent in the crystal structure of the CDK9/cyclin T1/Tat complex. The C-terminal part of Tat (residues 49–86) is not resolved in the crystal structure indicating that this part of the Tat is likely to be unstructured and also exposed to solvent. This is further supported by the analysis of the last 37 C-terminal residues of Tat (RKKRRQRRRAHQNSQTHQVSLSKQPTSQPRGDPTGPKE) that showed no identifiable sequence motifs to form secondary structures and was predicted to be a random coil by two independent protein secondary structure prediction servers (AGADIR: http://agadir.crg.es/ and JPRED: http://www.compbio.dundee.ac.uk/www-jpred/). In contrast, the N-terminal part of Tat that includes Ser-16 and Ser-46 residues was found to be quite stable in the CDK9/cyclin T1/Tat complex during the 20 ns MD simulations, preserving overall fold and correct coordination of Zn^2+^ ions and therefore could be used as a template for comparative analysis of possible structural consequences of Ser-16 and Ser-46 phosphorylation.

Phosphorylation of Ser-16 residue resulted in the relocation of its side chain from the internal interface position to the external solvent exposed position, disruption of a hydrogen bond between Tat Ser-16 and cyclin T1 Val-54 (Fig. 9b) and formation of a hydrogen bond between Ser-16 phosphate group and the first methionine of Tat (Fig. 9c). The transition also led to the formation of a hydrogen bond between Tat Glu-9 and CDK9 Lys-144 (Fig. 9d, e). In order to quantitatively characterize the extent of this transition, we calculated Solvent Accessible Surface Area (SASA) of the PO_3_ group of Tat Ser-16. The calculations showed that this transition takes only 5 ns when SASA of the PO_3_ group changed from insignificant 3 Å^2^ to a very significant 51.2 Å^2^. During the rest of the 20 ns MD trajectory, SASA of the PO_3_ group fluctuated around ~ 50 Å^2^. This solvent exposed conformation of phosphorylated Ser-16 was stabilized by strong hydrogen bond that formed between Ser-16 PO_3_ and Tat methionine (Fig. 9c) as mentioned above.

Another consequence of Ser-16 phosphorylation was the relocation of Tat segment (amino acid residues 17–34) that coordinates the two bound Zn^2+^ ions. As shown in Fig. 9, during this transition the Zn^2+^ ions preserved their coordination partners (Tat’s Cys-22, Cys-25, Cys-30, Cys-34, Cys-37 and Cyclin T1’s Cys-261), but changed their positions relative to CDK9 and cyclin T1. Although, Cα Root-Mean-Square Deviation (RMSD) of this segment at 20 ns MD time in comparison with the initial conformation is almost identical for S16&S46 and S16P&S46P complexes (0.7Å and 0.8Å respectively), the positions of the Zn^2+^ ions were shifted from their initial crystal structure positions by more than 4 Å indicating rigid body-like rotation of the Tat segment due to relocation of phosphorylated Ser-16 side chains from the internal to the external position. Analysis of large scale flexibility of the CDK9/cyclin T1/Tat complexes in non-phosphorylated and phosphorylated states conducted with DynDom v. 1.5 [19] showed the presence of two flexible domains in Tat structure (residues 5–17 and 18–25 & 29–37). During 20 ns MD the second flexible domain (residues 18–25 & 29–37) of Tat in S16P&S46P complex rotated as a rigid body by 33.2° as compared to the position of the domain in S16&S46 CCT complex. Tat’s Gln-17 acted as a mechanical hinge residue of this flexible domain. Since the Zn^2+^ binding segment of Tat protein in the CDK9/cyclin T1/Tat complex is externally accessible, and it is likely to interact with other proteins involved in HIV-1 transcription activation such as the components of super elongation complex. Therefore its relocation in activated CDK9/cyclin T1/Tat complex might have a significant effect on HIV-1 transcription.

## Conclusion

**Fig. 10:**
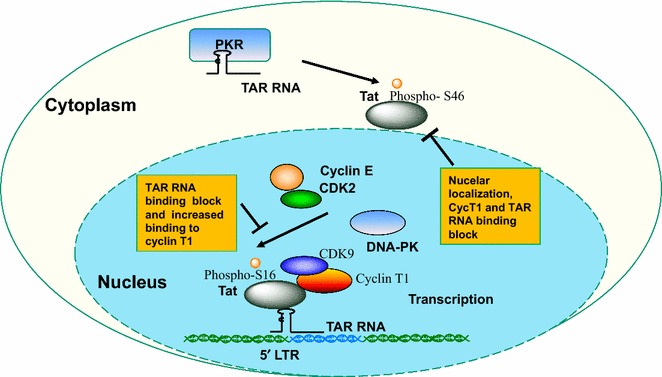
Schematic representation of the effect of Tat phosphorylation on HIV-1 transcription regulation. CDK2/cyclin E or DNA-PK phosphorylates Tat Ser-16 which facilitates binding to TAR RNA and reduces the interaction with CDK9/cyclin T1. PKR phosphorylates Tat Ser-46 which may affect Tat nuclear localization and prevents Tat binding to cyclin T1

Overall, our findings showed that HIV-1 Tat is phosphorylated on Ser-16 residue in cultured cells (summarized in Fig. 10). CDK2/cyclin E and to a lesser extent DNA-PK is likely to phosphorylate Tat Ser-16 and induce HIV-1 transcription and replication. In contrast, PKR that phosphorylates primarily Tat Ser-46 inhibits HIV-1 replication, as shown previously [11] and as our PKR knock down experiment in this study pointed out. Thus, phosphorylated Tat Ser-16 and Tat Ser-46 residues seem to play distinct regulatory roles. Ser-16 phosphorylation can facilitate Tat binding to TAR RNA and may facilitate rearrangement of CDK9/cyclin T1/cyclin T1 complex and help to dissociate CDK9/cyclin T1 from TAR RNA bound Tat during transcription elongation. In contrast, Tat Ser-46 phosphorylation prevents Tat localization to the nucleus and reduces binding to TAR RNA and cyclin T1, thus imposing overall transcription block for Tat function. Our findings presented here indicate a novel regulatory mechanism of HIV-1 transcription mediated by Tat Ser-16 phosphorylation.

## Discussion

Latent infection prevents complete HIV-1 eradication by antiretroviral drugs that are only effective against actively replicating HIV-1 virus. On the other hand, activation of HIV-1 transcription is not affected by the existing antiretroviral drugs and continuous virus expression in residual macrophages may lead to HIV-1 associated pathogenesis [20]. Many factors contribute to the establishment of latency, including inefficient transcription activation by Tat that may be the result of its insufficient expression, activity of cellular cofactors or absence or modification of Tat itself [1]. Tat recruits P-TEFb from the high molecular weight complex where kinase-inactive CDK9/cyclin T1 is bound to 7SK RNA, hexamethylene bis-acetamide-inducible protein 1 (HEXIM1) dimer, Lupus antigen-related protein 7 (LARP7) protein [21–23] and the methylphosphate capping enzyme (MePCE) [24, 25]. Tat also facilitates the formation of super elongation complex (SEC) that contains additional elongation factors and co-activators, including AFF4, ELL2, AF9, ENL [26–28]. AFF4 acts as a scaffold for SEC assembly interacting with cyclin T1 [26]. Tat, on the other hand, increases protein level of ELL2 that is targeted by Siah1 for polyubiquitination and degradation that stabilizes SEC [26–28]. Tat may also recruit ELL1 forming a distinct SEC complex [28]. In a future study, it will be interesting to determine whether Tat Ser-16 phosphorylation promotes Tat association with SEC and/or SEC stabilization. We found here that phosphorylated Tat Ser-16 residue may disrupt a hydrogen bond between Tat Ser-16 and cyclin T1 Val-54 and form a hydrogen bond between Tat Glu-9 and CDK9 Lys-144. Thus, Tat Ser-16 phosphorylation can potentially rearrange CDK9/cyclin T1/Tat complex that may affect the association with SEC. Interestingly, Tat Ser-16 phosphorylation also reduces cyclin T1 binding. One can speculate that non-phosphorylated Tat recruits CDK9/cyclin T1 to TAR RNA, where Ser-16 phosphorylation facilitates its binding to TAR RNA but also loosens the association with CDK9/cyclin T1 which may help to dissociate P-TEFb from TAR RNA during transcription elongation.

We showed here that Tat is phosphorylated in cultured cells primarily on Ser-16 residue located within the N-terminal activation domain. While we detected the presence of Ser-46 phosphopeptide, the signal strength was comparably low and thus the confidence of Ser-46 phosphorylation detection was also comparably low. Thus we are not able to definitely conclude whether Ser-46 is phosphorylated in cultured cells. We also detect with low confidence additional phosphorylation sites located in the C-terminus, so the importance of these additional sites needs to be further investigated. Hence, in vivo, the Tat Ser-16 residue is likely to be the major phosphorylation site. In vitro, Tat Ser-16 can be phosphorylated by CDK2/cyclin E as shown here and also by DNA-PK as shown previously by Tyagi’s lab [6] whereas Ser-46 was phosphorylated by PKR. We also tested small molecule inhibitors of CDK2 and DNA-PK which led to ~ 30 and ~ 15% reduction in Ser-16 phosphorylation, correspondingly. Thus Tat Ser-16 is likely to be controlled by at least two distinct kinases in vivo. It will be interesting to combine CDK2 and DNA-PK inhibitors and test them in vivo, for example in humanized HIV-1 infected mice. All these observations are in agreement with our earlier study in which CDK2 was proposed to phosphorylate Ser-16 [4] and a recent study by Yong-Soo Bae and colleagues who showed that Tat is phosphorylated by PKR on Thr-23, Thr-40, Ser-46, Ser-62 and Ser-68 in vitro [11]. Also in agreement with our recent study we showed that CDK2 knock down inhibited HIV-1 replication [15], whereas PKR knock down induced it in agreement with Yong-Soo Bae and colleagues study [11].

Our previous work showed that CDK2/cyclin E plays a key role in HIV-1 transcription [3, 29]. We reported that CDK2 phosphorylates CDK9 on Ser-90 residue located within (^90^SPYNR^94^) consensus phosphorylation site and that alanine mutation of Ser-90 inhibited HIV-1 transcription [30]. Recently, CDK2 was also shown to phosphorylate SAM domain and HD domain-containing protein 1 (SAMHD1) that controls the cellular deoxyribonucleoside triphosphate (dNTP) pool sizes by hydrolyzing dNTP and inhibits HIV-1 reverse transcription [31–33]. Our recent study showed that CDK2 activity was inhibited in peripheral blood mononuclear cells obtained from patients with sickle cell disease, which led to the activation of SAMHD1 and inhibition of ex vivo HIV-1 infection [15]. Our current findings point to Tat Ser-16 as an additional relevant targets for CDK2 phosphorylation in HIV-1 replication. Thus, CDK2 remains to be a plausible kinase for future anti-HIV-1 therapeutics.

We showed here that HIV-1 transcription was inhibited when Ser-16 residue was mutated to alanine, but the transcription was almost fully recovered with Tat S16E mutation. This finding suggests that Ser-16 phosphorylation has a positive regulatory effect in HIV-1 transcription and viral replication. As pointed above, this could be due to enhancement of the binding to TAR RNA and changes in association of Tat with P-TEFb. In contrast, Tat S46A mutation was clearly inhibitory and Tat S46E mutation did not recover HIV-1 transcription. However, we were able to assemble a recombinant virus with Tat S46E mutation but it replicated poorly comparing to the WT Tat and Tat S16E viruses suggesting that Ser-46 phosphorylation can suppress viral replication. This conclusion is in agreement with Yong-Soo Bae and colleagues report who showed that Tat Ser-46 phosphorylation prevents Tat shuttling to the nucleus [11]. Accordingly, we observed retention of Tat S46E mutant in the cytoplasm. Thus, inhibitory mechanism of Ser-46 phosphorylation includes defects in Tat translocation and binding to TAR RNA and CDK9/cyclin T1 in the agreement with previous reports [11]. Tat is monoubiquitinated by Hdm2 in a non-proteolytic fashion on Lys-71 residue [13] which we explored here in conjunction to the Tat phosphorylation. However, we did not detect any strong effect of Tat Ser-16 mutations and only a weak effect of Tat S46D mutation on ubiquitination suggesting that Tat phosphorylation has no direct effect on Tat ubiquitination. The overall Tat phosphorylation was significantly reduced not only with Ser-16 mutation but also with Ser-46 alanine mutation. Thus it remains to be determined why Ser-46 affects the overall phosphorylation which primarily takes place on Ser-16.

Earlier studies showed that PKR phosphorylates C-terminus of Tat [7, 8, 10]. We previously demonstrated that short 57 nucleotide-long TAR RNA inhibited PKR, while the longer 82 nucleotide-length TAR RNA activated PKR [34]. Thus PKR can be both inhibited and activated in HIV-1 transcribing cells and thus might participate in Tat phosphorylation and deregulation of HIV-1 replication. Yong-Soo Bae and colleagues showed that p53 suppressed HIV-1 replication through the activation of PKR [11]. In their study, PKR-mediated phosphorylation prevented Tat from translocation to the nucleus and inhibited its interaction with TAR RNA and CDK9/cyclin T1, in agreement with our findings. Since Yong-Soo Bae and colleagues only analyze Tat phosphorylation by PKR in vitro and confirmed Tat phosphorylation with a mutation analysis and phospho-threonine and phospho-serine specific antibodies [11], it remains to be determined if Tat is phosphorylated in vivo by PKR under the conditions when PKR is activated. As mutations in both Tat Ser-16 and Tat Ser-46 downregulate the overall Tat phosphorylation level, mutation analysis is clearly not sufficient and direct phosphorylation analysis is needed to detect Ser-46 phosphorylation. PKR was shown to be inhibited by several mechanisms during HIV-1 replication that include the inhibition by TAR RNA, TRBP, adenosine deaminase ADAR1 and PKR regulatory factor PACT (see review [35]). PKR is also inactive in some cultured cell lines including Jurkat T cells [36], which were used in some experiments by Yong-Soo Bae and colleagues. While in their study binding of Tat to TAR RNA was reduced with 23–40–46–62–68 D mutations, single Tat S46D mutation had less pronounced effect on the binding of Tat to TAR RNA [11]. This is in agreement with our findings here that depicted a minimal effect of Tat S46D mutation on the binding to TAR RNA which was only strongly affected by Tat S16A mutation. Yong-Soo Bae and colleagues showed that HIV-1 provirus with S46A mutation was defective in viral replication [11], providing a support to our current observations that pNL4-3 proviruses with Tat S16A or Tat S46A mutations were inactive.

Our analysis of Tat binding to cyclin T1 showed that both Ser-16 and Ser-46 aspartic acid mutations reduced the binding. While strong reduction of the binding for aspartic acid mutants was unexpected, the Tat S16E mutant is likely to retain some activity as it is able to replicate. It is also possible that phosphomimetic mutations do not fully capture the effect of physiological phosphorylation which can be dynamic.

The analysis of CDK9/cyclin T1/Tat complex in silico by MD simulations showed that Ser-16 was embedded in the Tat-cyclin T1 interface and formed a hydrogen bond with cyclin T1’s Val- 54, thus potentially stabilizing the structure of CDK9/cyclin T1/Tat complex. This hydrogen bond was preserved during the MD simulation suggesting its high stability. Unlike Ser-16, the Ser-46 residue was found to be exposed to solvent in the crystal structure of the CDK9/cyclin T1/Tat complex. Phosphorylation of Ser-16 residue resulted in the disruption of a hydrogen bond between Tat Ser-16 and cyclin T1 Val-54 and formation of a hydrogen bond between Tat Glu-9 and CDK9 Lys-144. This relocation was relatively rapid taking only 5 ns out of the total 20 ns MD simulation. The solvent accessible surface area (SASA) was increased by more than factor 10 for the Ser-16 phosphate group. Also, Ser-16 phosphorylation was found to relocate the Zn^2+^ ions-binding Tat segment (amino acids 17–34), which is likely to affect the overall stability of the CDK9/cyclin T1/Tat complex. Large scale flexibility analysis of CDK9/cyclin T1/Tat complex showed that in S16P&S46P Tat, flexible domains (residues 18–25 and 29–37) were rotated along the Gln17 residue acting as a hinge by more than 30° resulting in a large shift of the Zn^2+^ ions. This rotation was not seen in the S16&S46 complex or S16&S46P complexes. Thus, the modeling data indicate that phosphorylated Ser-16 might stabilize CDK9/cyclin T1/Tat complex, while an alanine mutation of Ser-16 destabilized the CDK9/cyclin T1 interface.

Tat binds to the proteasome-associated PAAF1 factor, and modulates the proteasome function by switching it to the non-proteolytic mode [37]. Methylation of Tat’s Arg-52 and Arg-53 located in the TAR RNA binding domain of Tat reduces its binding to TAR RNA and P-TEFb and inhibits HIV-1 transcription [38]. Affinity of Tat to TAR RNA and P-TEFb is also increased with the acetylation of Tat’s Lys-28 [13]. We did not detect Tat methylation or acetylation as this domain of Tat is cleaved by trypsin and an additional enzyme is needed to recover this peptide for the MS analysis. Thus it remains to be determined whether Tat methylation or acetylation of its TAR RNA binding domain is related to Tat phosphorylation.

Taken together, our study identified Tat Ser-16 as a novel phosphorylation site that affected the overall Tat phosphorylation and regulated Tat interaction with TAR RNA and CDK9/cyclin T1. Thus, phosphorylation of Tat Ser-16 residue represents a novel mechanism of HIV-1 regulated transcription that may provide novel insights for a strategy to control HIV-1 transcription from latent HIV-1 provirus.

## Methods

### Materials

293T cells were purchased from ATCC (Manassas, VA). Anti-FLAG monoclonal antibodies were purchased from Sigma (St. Louis, MO) and protein A/G agarose beads from Santa Cruz Biotechnology (Santa Cruz, CA). Recombinant CDK9/cyclin T1 and CDK2/cyclin E were purchased from ProQinase (Freiburg, Germany). GST-tagged truncated recombinant human PKR (EIF2AK2, amino acids 252–551) was purchased from Thermo Fisher (Waltham, MA). Double-stranded RNA (polyinosinic-polycytidylic acid) was purchased from Sigma (St. Louis, MO). Horseradish peroxidase (HRP)-conjugated F(ab)_2_ fragment was purchased from GE Healthcare (Piscataway, NJ). All other inorganic reagents were purchased from Fisher Scientific (Fair Lawn, NJ) or Sigma. Radioactive materials were purchased from Perkin-Elmer (Waltham MA).

### Plasmids

The HIV-1 genomic vector, pNL4-3.Luc.R^−^E^−^ (Courtesy of Prof. Nathaniel Landau, NYU School of Medicine, New York, NY) was obtained from the NIH AIDS Research and Reference Reagent Program. pAd.CMV link 1 Tat plasmids expressing WT Tat, TatS16A, Tat S16D, Tat S46A and Tat S46D were generated as previously described [4]. pCI-His-hUbi plasmid was obtained from AddGene (Cambridge, MA).

### Knockdown of PKR and CDK2

Lentiviruses expressing small hairpin RNA (shRNA)-targeting human PKR, CDK2, and control shRNA were purchased from Santa Cruz (Dallas, Texas). CEM T cells were infected at 1.1 MOI/1000 cells. Spinoculation was carried out at 800×*g* for 30 min. Cells were then incubated for 24 h prior to the addition of puromycin (0.75 μg/ml) for selection of the shRNA-expressing clones. Efficiency of knockdown was assessed by real-time PCR analysis. Total RNA was extracted using TRIzol reagent according to the manufacturer’s protocol (Invitrogen, Grand Island, NY). Total RNA (100 ng) was reverse-transcribed to cDNA using Superscript™ RT-PCR kit (Invitrogen, Carlsbad, CA), hexamers and oligo-dT were used as primers. For real-time PCR analysis, cDNA was amplified using Roche LightCycler 480 and SYBR Green1 Master mix (Roche Diagnostics, Indianapolis, IN). PCR was carried out with denaturation at 95 °C for 10 s, annealing at 60 °C for 10 s, and extension at 72 °C for 10 s for 45 cycles. The 18S rRNA was used as a house keeping normalization standard for quantification of mRNA levels of PKR and CDK2. The following primers were used: PKR forward, CAAGTAAAGATTGGAGACTTTGGA; PKR reverse, TCAAATCTGTACCGCCGAAT; CDK2 forward, TTTGCTGAGATGGTGACTCG; CDK2 reverse, CTTCATCCAGGGGAGGTACA; 18S rRNA forward, CTGTTGCTACATCGACCTTT; 18S rRNA reverse, CTCCAGGTTTTGCAACCAGT. Mean Cp values for target genes and 18S rRNA were determined and ΔΔCt method was used to calculate relative expression levels.

### HIV-1 infection assays

Vesicular stomatitis virus G protein (VSV-G)**-**pseudotyped pNL4-3.Luc.R-E-virus (HIV-1-LUC-G) was prepared as previously described [39]. CEM-T cells were infected and cultured at 0.5 × 10^5^ cells/ml in 96-well plate at 37 °C and 5% CO_2_ for 48 h. The cells were collected, washed with PBS and resuspended in 100 μl PBS. Then, 100 μl reconstituted luciferase buffer (Luclite Kit, Perkin Elmer) was added to each well and after 10 min incubation, the lysates were transferred into white plates (Perkin Elmer) and luminescence measured using GloMax luminometer (Promega).

### p24 ELISA

CEM T cells were infected with pseudotyped viruses pNL4-3.Luc.R-E with WT Tat and Tat S16A, S16E, S46A and S46E mutants. The supernatant was collected 48 h post- infection and p24 protein was detected using RETRO-TEK/ZeptoMetrix HIV-1 p24 ELISA kit (Cat. #0801200). HIV-1 p24 antibody coated microplate was prewashed with a washing buffer, then added 200 µl of samples and p24 antigen standards (125, 62.5, 31.3, 15.6, 3.9 and 0 pg/ml) and incubated overnight at 37 °C. The plate was then washed and 100 µl of HIV-1 p24 detector antibody was added to each well and incubated for 1 h at 37 °C. The plate was again washed, then added 100 µl of Streptavidin-Peroxidase and incubated for 30 min at 37 °C. A blue color was developed within 20 min at room temperature, following the addition of 100 µL of substrate solution. The color development was stopped and the optical density was read at 450 nm using the iMark microplate reader (Bio Rad). The standard curve derived from the reading was used to interpolate the concentration of HIV-1 p24 protein in each sample.

### TAR RNA design

Biotinylated WT TAR RNA (59 nt) and mutant TAR RNA with the deletions of stem-loop nucleotides 21–27 and 38–41 were synthesized by Integrated DNA Technologies (Coralville, Iowa).

### Tat Mutagenesis in pNL4-3.Luc.R-E- and pCMV Link 1 Flag-Tat vectors

QuikChange XL site-directed mutagenesis kit (Agilent, Santa Clara, CA) was used to generate mutants of Tat. To mutagenize Tat in HIV-1 proviral pNL4-3.Luc.R^−^E vector, Sal1-BamH1 fragments were excised and subcloned in pEGFP-N1 vector (Clontech, Mountain View, CA). Tat Ser-16 residue was mutated to alanine with GCCCTGGAAGCATCCAGGAGCTCAGCCTAAAACTGCTTGTACC (forward) and GGTACAAGCAGTTTTAGGCTGAGCTCCTGGATGCTTCCAGGGC (reverse) primers and to glutamic acid with the GCCCTGGAAGCATCCAGGAGAACAGCCTAAAACTGCTTGTACC (forward) primer and GGTACAAGCAGTTTTAGGCTGTTCTCCTGGATGCTTCCAGGGC (reverse) primer. Tat Ser-46 residue was mutated to alanine with GACAAAAGCCTTAGGCATCGCCTATGGCAGGAAGAAGCGG (forward) and CCGCTTCTTCCTGCCATAGGCGATGCCTAAGGCTTTTGTC (reverse) primers and to glutamic acid with GACAAAAGCCTTAGGCATCGAATATGGCAGGAAGAAGCGG (forward) and CCGCTTCTTCCTGCCATATTCGATGCCTAAGGCTTTTGTC (reverse) primers. PCR reactions were run for 18 cycles with the extension time of 8 min to allow the synthesis of the whole plasmid sequence. PCR products were digested with *Dpn I* to degrade the original template. The PCR products were transformed into XL-Gold cells. Colonies were picked, and DNA was isolated using High Pure Plasmid Isolation kit (Roche Applied Sciences, San Francisco, CA). The obtained clones were sequenced using service from Macrogen (Rockville, MD). To reconstruct HIV-1 provirus, Sal1-BamH1 DNA fragments contacting Tat mutant sequences were subcloned back to pNL4-3.Luc.R^−^E vector.

Mutagenesis of Tat in expression pCMV Link 1 vector was done directly in the target vector using the same procedure described above. Tat Ser-16 residue was mutated to alanine with TGGGAGCATCCAGGAGCTCAGCCTAAGACTGCT (forward) and AGCAGTCTTAGGCTGATCTCCTGGATGCTCCCA (reverse) primers and to aspartic acid with the TGGGAGCATCCAGGAGATCAGCCTAAGACTGCT (forward) primer and AGCAGTCTTAGGCTGATCTCCTGGATGCTCCCA (reverse) primer. Tat Ser-46 residue was mutated to alanine with AAGGCTTAGGCATCGCCTATGGCAGGAAGAAG (forward) and CTTCTTCCTGCCATAGTCGATGCCTAAGCCTT (reverse) primers and to aspartic acid with AAGGCTTAGGCATCGACTATGGCAGGAAGAAG (forward) and CTTCTTCCTGCCATAGTCGATGCCTAAGCCTT (reverse) primers. To mutate Tat lysine 71 which is the subject of ubiquitination, an alanine mutant (K71A) was generated in pCMV Link 1 Tat vector using TCAGACTCATCAGGCTTCTCTATCAGCGCAATCCCTACCC (forward) and GGGTAGGGATTGCGCTGATAGAGAAGCCTGATGAGTCTGA (reverse) primers following the above mentioned procedure.

### Transfections

293T cells were seeded in 6-well plates to achieve 50% confluence at the day of transfection. The cells were transfected with indicated plasmids using Lipofectamine and Plus reagents (Life Technologies) following manufacturer’s protocol. The efficiency of transfection was verified using a plasmid encoding green fluorescent protein. The cells were cultured for 48 h posttransfection and then analyzed.

### Immunoprecipitations

293T cells were lysed in whole cell lysis buffer (50 mM Tris–HCl, pH 7.5, 0.5 M NaCl, 1% NP-40, 0.1% SDS) supplemented with protease cocktail. Tat was precipitated as indicated with anti-Flag antibodies and as we previously described [4]. Briefly, 400 μg of lysate and 800 ng of antibodies were combined with 50 μl of 50% slurry of protein A/G agarose and incubated for 2 h at 4 °C in a TNN Buffer (50 mM Tris–HCl, pH 7.5, 150 mM NaCl and 1% NP-40). The agarose beads were precipitated and washed with TNN buffer, resolved on 12% Tris-Tricine SDS-PAGE, transferred to polyvinylidene fluoride (PVDF) membranes and immunoblotted with appropriate antibodies. In case of in vivo Tat phosphorylation experiments the gel was dried and exposed to Phosphor Imager screen.

### Phosphorylation of Tat-derived peptides in vitro

Tat-derived peptides were synthesized by GenScript (Piscataway, NJ). About 2 µg of peptides Tat (12–29) ^12^HPGS^16^QPKTACTPCYCKK^29^ containing Tat Ser-16 (dissolved in 1 mM DTT and sonicated prior to reaction); Tat (29–45) ^29^KCCFHCQVCFTTKGLGI^45^ (sonicated prior to reaction); Tat (41–57) ^41^KGLGIS^46^YGRKKRRQRRR^57^ containing Tat Ser-46; and Tat (57–71) ^57^RAPQDSQTHQASLSK^71^ (sonicated prior to reaction) were phosphorylated by recombinant human CDK2/cyclin E, CDK9/cyclin T1 and truncated recombinant human PKR (PKR reaction mix contained 10 ng of dsRNA) in a 10 μl reaction containing 50 mM Hepes–KOH buffer (pH 7.5), 10 mM MgCl_2_, 6 mM EGTA, 2.5 mM DTT, 100 µM cold ATP and 5 µCi γ-(^32^P)ATP and incubated for 30 min at 30 °C. The reactions were stopped with Laemmli sample buffer containing 6 M urea, resolved on 12% Tris-Tricine SDS-PAGE with 6 M urea, stained with SimplyBlue safe stain (Thermo Fisher) and subjected to autoradiography and quantification with Phosphor Imager (Packard Instruments, Wellesley, MA).

### Hunter phosphopeptide mapping

Phosphopeptide mapping was conducted using Hunter thin layer peptide mapping electrophoresis system (C.B.S. Scientific, Del Mar, CA). Tat-derived peptides were phosphorylated by recombinant CDK2/cyclin E or recombinant PKR with (^32^P)ATP as described above. Phosphorylated peptides were applied to thin layer cellulose plates (Boehringer Mannheim, Indianapolis, IN) and separated by electrophoresis at pH 1.9 (H_2_O-acetic acid–formic acid, 900:78:22) conducted at 1000 V, 12 mA for 1 h. Cellulose plates were dried and stained with 0.25% ninhydrin in acetone. The plates were then exposed to Phosphor imager screen.

### HIV-1 Tat phosphorylation in vivo

293T cells were transfected with pAd.CMV link 1 Flag-Tat WT, Tat S16A or Tat S46A mutant expressing plasmids using Lipofectamine and Plus reagents (Life Technologies). After 48 h incubation, the cells were placed in a phosphate-free and serum-free DMEM media (Life Technologies) for 2 h. Subsequently, the media was changed to phosphate-free DMEM media supplemented with 0.5 mCi/ml of (^32^P)-orthophosphate and cells were further incubated for 3 h at 37 °C. To increase Tat phosphorylation, 0.1 μM okadaic acid (Sigma) was added to block cellular PPP-phosphatases. Cells were washed with PBS and lysed in whole cell lysis buffer containing 50 mM Tris–HCl, pH 7.5, 0.5 M NaCl, 1% NP-40, 0.1% SDS and protease inhibitors cocktail (Sigma). After 10 min on ice, cellular material was scraped, incubated at 4 °C for 30 min on a shaker and then centrifuged at 14,000 rpm, at 4 °C for 30 min. The supernatant was recovered and protein concentration was determined using Lowry protein assay (Bio-Rad). Tat was precipitated with monoclonal anti-Flag antibodies (Sigma) coupled to protein G-agarose for 2 h at 4 °C in a TNN Buffer (50 mM Tris–HCl, pH 7.5, 0.15 M NaCl, 1% NP-40). Tat was resolved on 12% Tris-Tricine SDS-PAGE. Tat containing bands were excised and subjected to in gel reduction, alkylation and digestion with trypsin as previously described [40].

To analyze the effect of CDK2 and DNA-PK inhibitors on Tat phosphorylation, 293T cells were grown to 40% confluence and transfected with Flag-Tat expression vector using Lipofectamine 3000/PLUS in OPTI-MEM media as directed by manufacturer. At 24 h posttransfection, the cells were treated overnight with 5 µM SU9516 (Tocris), which is an inhibitor of CDK2 with estimated in vitro IC_50_ = 22 nM. Parallel samples were also treated overnight with 2.5 µM NU7441 (Tocris), an inhibitor for PK DNA with estimated in vitro IC50 = 14 nM. Control cells were treated with DMSO. At 16 h posttreatment, all cells were additionally treated with 100 nM okadaic acid for 2 h. Tat was purified and processed for mass spectrometry analysis as describe above.

### Mass spectrometry

Samples were loaded onto in-house prepared nano C18 column and eluted for 60 min with 2–30% gradient of acetonitrile and flow rate 300 nl/min using Shimadzu Prominence Nano HPLC. The 1 FT MS scan and 3 data dependent FT MS/MS scans were performed on Thermo LTQ Orbitrap XL mass spectrometer on major multi-charged MS peaks with resolution 60,000 in each event set. Samples from each patient were run in triplicate. The resulting set of MS/MS spectra were analyzed by Proteome Discoverer 2.1 with SEQUEST (Thermo) search engine (precursor tolerance 30 ppm and fragments tolerance 0.1 Da). These high resolution data and search criteria reduce amount of false positives and dramatically decrease the search time. Protein identifications were carried out using Proteome Discoverer 1.4 software in combination with the SEQUEST protein database search engine and International Protein Index (IPI) Human Protein Database (version 1.79) to which HIV-1 proteins were added. A sequential database search was performed using the human FASTA database. Only peptides with a cross-correlation (XCorr) cutoff of 2.6 for [M + 2H]^2+^, 3.0 for [M + 3H]^3+^ and a higher charge state were considered. These SEQUEST criteria typically result in a 1–2% false discovery rate (FDR). The FDR was determined by searching on a decoy database. We used SIEVE 2.1 software (Thermo Fisher, Waltham, MA, USA) which is compatible with Proteome Discoverer 1.4 for label-free quantitative analysis of the high resolution MS spectra produced by Orbitrap MS scans.

### Flow cytometry

Cell suspensions were fixed and permeabilized using BD Biosciences kit (554714) followed by staining with primary antibodies against CDK2 (Santa Cruz, sc6248) or PKR (Santa Cruz, sc393038) and secondary anti-rabbit IgG antibody (FITC) from Invitrogen. Stained cells were analyzed with FACSCalibur instrument (Becton–Dickinson) and CellQuest software. Three independent experiments were carried out for each sample.

### Analysis of Tat–TAR RNA interaction

A slurry (40 μl) of streptavidin agarose beads (Invitrogen) was blocked for 30 min with 64 μg yeast tRNA and 100 μg BSA in Binding buffer (20 mM Tris–HCl pH 7.5, 2.5 mM MgCl_2_, 100 mM NaCl) and then incubated with 10 μg of biotinylated TAR RNA or biotinylated mutant TAR RNA on a rotating platform at 4 °C. Whole cell lysates from 293T cells grown in 6-well plates and transfected with 2 μg/well of plasmids expressing WT Flag-Tat, Flag-Tat S16A or Flag-Tat S46A were added to the beads in TAK buffer (50 mM Tris–HCl pH 8.0, 5 mM MgCl_2_, 5 mM MnCl_2_, 10 μM ZnSO_4_, 1 mM DTT, 100 mM NaCl) and rotated for 2 h at 4 °C. Beads were washed in TNN Buffer (50 mM Tris–HCl, pH 7.5, 0.15 M NaCl, 1% NP-40) and eluted in 2x SDS-loading buffer to resolve on 12% SDS-PAGE. Immunoblots were probed with anti-Flag and anti-biotin antibodies.

### Analysis of Tat ubiquitination

To analyze Tat ubiquitination in vivo, 293T cells grown in 6-well plates were co-transfected with 1 μg/well of pCI-His-hUbi plasmid (Addgene) and 1.5 μg/well of WT Flag-Tat, Flag-Tat S16A, Flag-Tat S16D, Flag-Tat S46A, Flag-Tat S46D or Flag-Tat K71A plasmid using Lipofectamine as described above. At 48 h posttransfection, the cells were washed twice in PBS, and resuspended in Guanidine denaturing buffer (6 M Guanidium-HCl, 100 mM Na_2_HPO_4_, 10 mM Imidazole and Sigma protease inhibitors). Cell lysates were sonicated and cleared by centrifugation. His–Ub conjugated proteins were purified on Ni–NTA agarose beads (Novagen, Madison, WI). Each sample lysate (400 μl) was combined with 50% slurry of Ni–NTA agarose beads (50 μl) and incubated at 4 °C for 4 h. The agarose beads were washed twice in Urea-containing buffer (100 mM NaH_2_PO_4_, 10 mM Tris Base, 8 M Urea and 10 mM Imidazol) and eluted with 2x SDS-loading buffer. Proteins were resolved on 12% Tris-Tricine SDS-PAGE, transferred to polyvinylidene fluoride (PVDF) membranes and immunoblotted with anti-Flag antibodies.

### Building a homology model of CDK9/Cyclin T1/Tat complex

Regularized models of the spatial structures of the CDK9/Cyclin T1/Tat complex were built based on the available crystal structures (PDB: 3MI9, 3MIA [17]) using homology modeling tools included in the standard protocols of the ICM-Pro software package (Molsoft LLC, USA) [41]. Loops absent in the crystal structures were built using the Monte-Carlo energy minimization protocol with the loop-modeling tools of ICM-Pro as described previously [30]. Only standard torsion angles (*φ*, *ψ*, *ω* and *χi*) of amino acids were allowed to vary during the energy minimization. The ICM default set of energy parameters (ECEPP/3 potential) for van der Waals, electrostatic, torsion energy interactions and hydrogen bonding were used in these calculations. Missing hydrogen and heavy atoms were added and atom types and partial charges were assigned. The protein models were adjusted so that the optimal positions of polar hydrogens were identified. Steric clashes were removed by the energy minimization. Amino acid point mutations in the protein structures and as well as phosphorylation of Ser and Thr residues were done using standard protocols of the ICM-Pro software package.

### MD simulations

MD simulations were performed using the double precision version of AMBER 12 (University of California, San Francisco), the widely used software package for molecular and dynamic modeling of proteins [42] running on the multiprocessor clusters of National Research Centre “Kurchatov Institute”. MD simulations involve several standard steps including: (a) creation of the protein topology file and preparation of input data for AMBER 12 using the TLEAP tool; (b) construction of hydration models for the protein complex under investigation in a periodic water box with a minimal distance to the water box border of 12 Å; (c) energy minimization and thermodynamic equilibration of hydrated proteins and surrounding solvent; (d) MD simulations at a constant temperature using the Amber99SB parameter set and the TIP3P water model [43, 44]. The thermodynamically equilibrated system was used to perform MD simulations at 310 °K using the Langevin thermostat with constant pressure (1 atm) and an MD duration of 20 ns with time steps of 2 fs. States of the model system were recorded after every 10 ps of MD time for analysis. Neighbor searching was performed every 10 steps. The PME algorithm was used for electrostatic interactions with a cut-off of 1.0 nm as implemented in AMBER. A cut-off of 1.0 nm was used for van der Waals interactions. SHAKE algorithm was used to constrain bonds involving hydrogen [45]. Large-scale flexibility of model proteins was analyzed using the DynDom v.1.5 software package [19].
